# 

**Published:** 1916-12-02

**Authors:** 


### Gifts to Hospitals.

Lord Mexborough has left £1,000 to the Battersea Anti-Vivisection Hospital.

Mr. Laurence Macdonald, of Carlisle, left a bequest of £250 to the Cumberland Infirmary, Carlisle.

Mrs. Elsie Ogston Watson Jackson, of Thorngrove, Mannofield, Aberdeen, who died on August 31, left £100 each to the Aberdeen Royal Infirmary and Aberdeen Dispensary.

The Huddersfield Royal Infirmary has received from Mrs. F. W. Sykes, of Green Lea, Hindley, a gift of £1,000, which by special request is to be used for general purposes.

Miss Ellen Sanderson, of Scarborough, whose estate has been proved at £14,321 gross value, left the residue of her property, after providing for certain legacies and bequests, between the Yorkshire County Hospital and the Scarborough Hospital.

Second-Lieut. Herbert Edward Evatt Coleman, Sussex Regiment, of Bexhill-on-Sea, who was killed in France on September 9 last, bequeathed £5,000 to St. Thomas's Hospital.

Under the will of Mr. Joseph Barrow, of Edgbaston, the Birmingham General Hospital Samaritan Fund and the Edgbaston Deaf and Dumb Institution will each receive a legacy of £100, free of duty.

Perhaps the most curious gift to an institution which has been acknowledged for some time is that of two sausages for each patient once a week—a gift one of the Willesden military hospitals has taken advantage of for two years.

Mr. W. P. Lowrie, J.P., of Bearsden, N.B., left £31,000 to hospitals, including £10,000 to the Royal Infirmary, £10,000 to the Western Infirmary, £3,000 to the Victoria Hospital, and £3,000 to the Royal Sick Children's Hospital, all at Glasgow.

The East Riding of Yorkshire County Council is conducting negotiations with the committees of the Hull Infirmary and the York and Scarborough Hospitals with a view to co-operating in a scheme for the treatment of venereal diseases. It is hoped to come to terms with Leeds University in respect of the bacteriological part of the work.

### Matrons' and Sisters' Appointments.

Porthcawl, St. John Auxiliary Military Hospital.—Miss M. H. Ruffle has been appointed matron. She was trained at the London Hospital, where she was afterwards massage sister, and has since been sister at Broseley Hospital, Salop; assistant matron at the Women's Hospital, Liverpool; matron of the Cottage Hospital, Malmesbury; of the Cottage Hospital, Wellington; and of the Rest Convalescent Home, Southerndown, Glamorganshire.

Blackpool, Station Road Hospital.—Miss Gertrude Farnworth has been appointed sister. She was trained at Townley Hospitals, Bolton, where she was later sister. She has also been school nurse under the Farnworth Education Committee.

Leasowe Hospital for Crippled Children. — Miss Katherine Rowland has been appointed ward sister. She was trained at Liverpool Royal Infirmary, and has since been sister at the Royal Southern Hospital, Liverpool, and Birkenhead Children's Hospital.

Penmore Hospital, Chesterfield.—Miss S. M. Rogers has been appointed sister. She was trained at St. George's Hospital, and has since been sister at the City Hospital, West Heath, Birmingham, and has done military nursing.

Samaritan Free Hospital.—Miss C. Tompson and Miss C. Dickson have been appointed sisters. Miss Tompson was trained at St. Bartholomew's Hospital, where she was senior theatre nurse, and has been sister at Haslar Hospital. Miss Dickson was trained at the West London Hospital, Hammersmith, and has since been staff nurse at Queen Charlotte's Hospital, and night sister at Hampstead General Hospital.

Romsley Sanatorium, Birmingham.—Miss Julia Lund has been appointed night superintendent. She was trained at Keighley Union Infirmary, and has been sister at Grimsby Union Infirmary, at Rotherham Union Infirmary, and at Park Hill Hospital, Dingle. Miss Lund has also done private nursing.

Ventnor, Royal National Hospital.—Miss Louise J. Strange has been appointed home sister, and Miss Ada L. Gough sister. Miss Strange was trained at Camberwell Infirmary, and has been sister and night superintendent at the Royal National Hospital, Ventnor; she has also done private nursing. Miss Gough was trained at Dudley Infirmary, and has since been sister at Ipswich Sanatorium and night sister at Romsley Hill Sanatorium, Birmingham.

**NOTE.—Particulars of Appointments for insertion in this column will be welcomed.**

### Personalia.

The Cheshire Education Committee has appointed Dr. Jean McLeod, M.D., Edinburgh, as assistant school medical officer for the county, at a salary of £350 per annum.

Mr. F. J. Alban, deputy accountant of the Welsh National Insurance Commission, was last week appointed, out of thirty-three candidates, to the post of chief accountant and controller to the Welsh National Memorial Association. The salary attached to the position is £500 a year.

Dr. D. M. Moffatt, R.A.M.C., whose name is contained in the latest list of honours as a recipient of the Military Cross, was formerly house surgeon at the Bootle Borough Hospital, and later became school medical officer under the Bootle Corporation.

RATES OF SUBSCRIPTION: Twelve months. 6/6; Six months, 4/-; Three months, 2/-; post free to any address in the United Kingdom. Abroad, Twelve months, 8/8: Six months, 5/-; Three months, 2/6, post free. THE HOSPITAL "Index" to Volume LX., April to September 1916, is now ready, price 2½d. per copy, post free. Address The Manager, "THE HOSPITAL," The Hospital Building, 28/29 Southampton Street, Strand, London, W.C.

